# Angiomatoid Fibrous Histiocytoma Mimicking a Lymph Nodal Lesion: A Case Report

**DOI:** 10.31729/jnma.5922

**Published:** 2022-02-28

**Authors:** Sailuja Maharjan, Bandana Satyal, Reena Baidya, Arbin Joshi, Pradeep Baral

**Affiliations:** 1 Department of Pathology, B&B Hospital Pvt. Ltd., Gwarko, Lalitpur, Nepal; 2 Department of Surgery, B&B Hospital Pvt. Ltd., Gwarko, Lalitpur, Nepal; 3 Department of Radiology, B&B Hospital Pvt. Ltd., Gwarko, Lalitpur, Nepal

**Keywords:** *angiomatoid fibrous histiocytoma*, *case report*, *groin*, *soft tissue neoplasm*

## Abstract

Angiomatoid fibrous histiocytoma is an uncommon soft tissue neoplasm with potential for recurrence and rare metastasis. The majority of cases are painless, slow growing and occur in superficial extremities of young adults. Here we report a case of Angiomatoid fibrous histiocytoma in a 28-year-old male patient presenting as a slowly growing painful mass in the groin region. This case is of particular interest due to its uncommon site of presentation and its misdiagnosis as lymph nodal lesion on radiology. Although it is a rare entity, it should be considered in differential diagnosis of soft tissue mass in a young patient.

## INTRODUCTION

Angiomatoid Fibrous Histiocytoma (AFH) is a rare soft tissue neoplasm accounting for 0.3% of all soft tissue tumors.^1^ AFH was first described by Enzinger in 1979 as angiomatoid malignant fibrous histiocytoma.^2-4^ It was classified under the category of intermediate tumour of uncertain differentiation as AFH by World Health Organization (WHO) in 2013.^5^ It commonly occurs in extremities in young individuals followed by trunk, head and neck.^6^ It has nonspecific clinical and radiological findings making its preoperative diagnosis very challenging.^3^ We present a case of AFH in an uncommon location which was misdiagnosed as a lymph nodal lesion on radiology.

## CASE REPORT

A 28-year-old gentleman presented with complaints of swelling in the left groin region for two months. The swelling was associated with pain and discomfort.

There were no other constitutional symptoms. The patient was previously operated on at another centre for swelling at the same site. The lesion then recurred after three years. It was diagnosed as a benign lesion, however, the previous biopsy report was unavailable.

**Figure 1:**
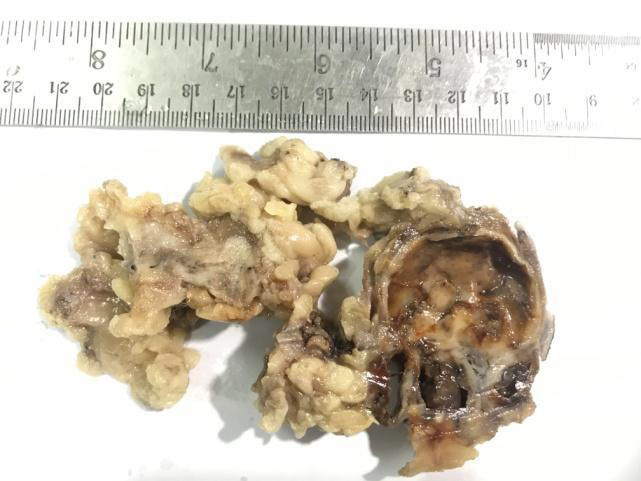
Gross specimen demonstrating psedoencapsulated solid-cystic tumor.

Contrast Enhanced Computed Tomography (CECT) of pelvis was done which showed a well-defined, lobulated partially cystic mass with enhancing solid component in the subcutaneous plane of left groin region lying lateral to the common femoral vessel and was given a provisional diagnosis of partially necrotic lymph nodal lesion. An excision biopsy at our centre revealed a skin-covered mass measuring 4.4x4x2.2cm (Figure 1).

**Figure 2:**
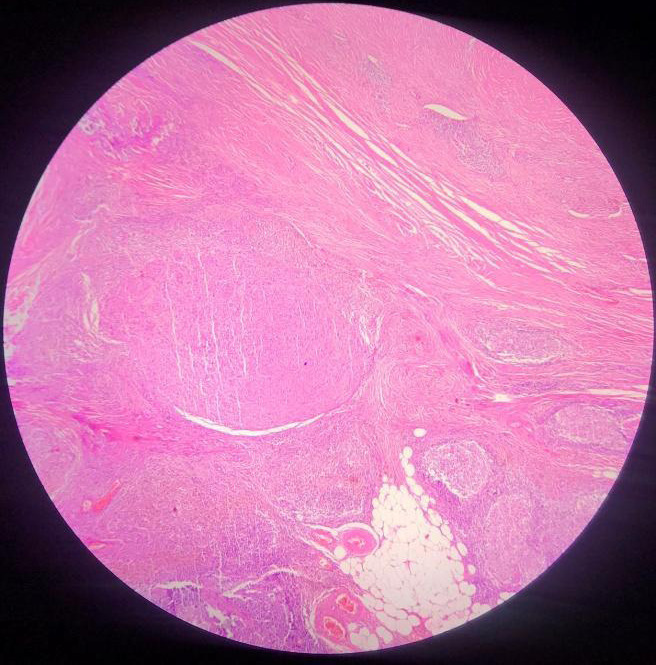
Histomorphology showing nodules of tumor cells with lymphoid follicles at periphery.

The mass was partially encapsulated with a cut section showing solid cystic areas with extensive haemorrhage. Multiple lymph nodes were identified, measuring 2.5x1.5x1cm. However, the skin surface was normal. Histopathological examination demonstrated an incompletely pseudo-encapsulated tumour in the deep dermis and subcutis comprising nodules of spindle-shaped cells arranged in sheets, bundles, whorls, and focally in storiform pattern (Figure 2).

**Figure 3:**
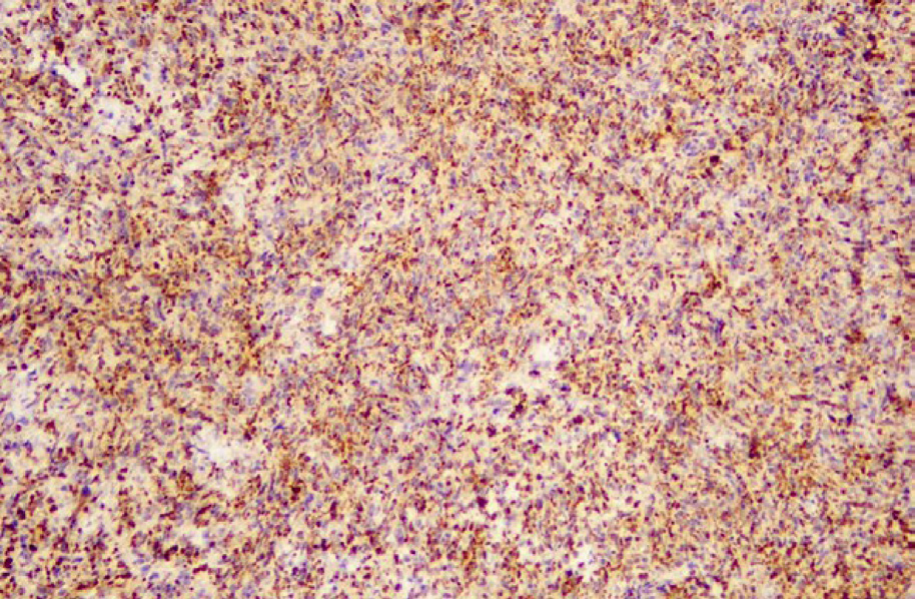
Immunohistochemistry showing CD68 positivity.

Large irregular pseudoangiomatous spaces were seen with extensive hemosiderin deposition. The focal area demonstrated pleomorphic cells with occasional mitotic figures. Lymphoid aggregates with germinal centre formation were frequently noted in the periphery of the tumour. The lymph nodes showed reactive features. The resected margins were negative. Based on the histomorphology, the preliminary diagnosis of angiomatoid fibrous histiocytoma was made and immunohistochemical evaluation was performed to further ensure the diagnosis and to rule out other tumours like a vascular tumour, melanoma, and other sarcomas. The results showed positive stains for vimentin, CD68, TLE1, CD99, and desmin (Figure 3). Proliferative marker Ki67 was <5%. CD34, CK, SMA, ERG, CD117, S100, BCL2, HMB45, Melan A, and STAT6 revealed negative stains.

## DISCUSSION

AFH is a rarely metastasizing intermediate grade mesenchymal neoplasm with an uncertain line of differentiation.^1^ It frequently presents in extremities as painless superficial mass followed by trunk and head and neck region.^2,7^ However, a history of pain was encountered in our patient. It has a predilection for the sites of normal lymphoid tissue such as axillary, inguinal, and supraclavicular regions.^3^ Occasionally, it can occur in extra somatic sites like the brain, lungs, retroperitoneum, bone, and breast.^8,9^ Most AFH presents during the first two decades of life although it can have wide age distribution.^6^ History of trauma has been elicited in a few cases.^7^ A handful of cases have experienced systemic symptoms like pyrexia, anaemia, and malaise attributed to intratumoral cytokine production.^2^ Its association with some paraneoplastic syndrome-like platelet storage pool deficiency has been mentioned in literature.^8^

It is very challenging to make an initial diagnosis of AFH due to its non-specific clinical and radiological findings. The possible differential diagnoses include diverse entities ranging from reactive to malignant tumours such as hematoma, hemangiomas, soft tissue sarcomas, and metastatic lymph nodes.^7^ Radiological evaluation suggested a partially necrotic lymph node in our case. Even though it is a rare neoplasm, it is very imperative to consider it in the differential diagnosis of soft tissue mass in young patients as the prognosis is excellent.

Consistent with the classic histomorphological picture of AFH reported in the literature, our case had all four features: a) multinodular growth of spindle to histiocytoid cells, b) blood-filled pseudoangiomatous spaces surrounded by tumour cells, c) incomplete fibrous pseudocapsule, d) peripheral lymphoplasmacytic infiltrate with the formation of the germinal center.^3,4^ Hence, devoting attention to these features are essential for the diagnosis. Additionally, there were some strikingly pleomorphic cells with mitotic figures in our case. But studies have exhibited that neither pleomorphism nor mitosis is correlated with clinical behavior.^5^

Furthermore, it can demonstrate a wide spectrum of morphological variations such as clear cell change, sclerosis, nuclear grooving, schwannoma-like pattern with nuclear palisading, rhabdomyoblast-like cells, small round cells with scant cytoplasm, pulmonary edema like pattern, reticular pattern, myxoid areas posing diagnostic dilemmas.^9,10^ The differential diagnosis considered with these heterogeneous patterns include schwannoma, aneurysmal fibrous histiocytoma, rhabdomyosarcoma, malignant extrarenal rhabdoid sarcoma, Ewing's sarcoma, myxoid fibrosarcoma, myoepitheliomas.^4,5^

AFH lacks a specific immunophenotype and has a supportive role only. Bohman SL, et al. reported that these tumours demonstrated immunoreactivity with desmin (56%) and CD68 (65%) which is consistent with our findings.^11^ Bruehl FK, et al. has reported in their study that an unusual combination of desmin and EMA can raise a high index of suspicion for AFH in an appropriate histomorphological background as it is also seen in a distinct entity of desmoplastic small round cell tumor.^8^ Likewise, CD99 expression has been documented in some studies which are again similar to our results.^2^ There is occasional expression of myeloid markers like SMA, calponin, h-caldesmon nevertheless, skeletal muscle markers are negative.^4,6^ Recently AFH has shown high expression of ALK.^8^ AFH is also indicated by negative expression of endothelial markers such as CD34, CD 31, STAT4 and other markers like CK, CD35, S100.^4^

AFH is now recognized as a translocation associated neoplasm and is associated with three translocations EWSR-CREB1, EWSR-ATF1, and FUS-ATF1.^5-7^ EWSR-CREB1 is the frequently described aberration which is documented in more than 90% cases but is not specific as it is also seen in other tumours like clear cell sarcoma, clear cell sarcoma like tumours of the gastrointestinal tract, hyalinizing clear cell carcinomas and primary pulmonary myxoid sarcomas.^9,10^

Studies have demonstrated the variability in the rate of local recurrence from 2-25% and is commonly observed in cases with infiltrative margins.^1,4^ As reported by Costa MJ, et al, its occurrence in extra somatic sites has shown to exhibit more recurrence rate and tend to be of larger size with high mean age compared to the somatic ones.^12^ There are no clinical, genetic, histomorphological or immunohistochemical parameters that can predict the behaviour of the tumor.^5^ The cumulative findings in a meta-analytical study have solicited that the metastatic rate is 8.7% although there are studies demonstrating metastasis in <1% which is predominantly in the regional lymph nodes.^2^

AFH is an intermediate-grade neoplasm that occurs commonly in the extremities of young adults. Since it is a rare tumour it can lead to erroneous diagnosis as there are no distinct clinical and radiological findings. The correct diagnosis of this entity is crucial as the vast majority of cases bear an excellent prognosis with a small risk of local recurrence and rare metastasis. Hence, wide excision with long-term clinical and radiological surveillance of the patient is highly warranted.
